# 2-(6-Bromo­benzo[*d*]thia­zol-2-yl)-5,5-di­methyl­thia­zol-4(5*H*)-one

**DOI:** 10.1107/S1600536813031334

**Published:** 2013-11-23

**Authors:** Hendryk Würfel, Helmar Görls, Dieter Weiss, Rainer Beckert

**Affiliations:** a Institut für Organische Chemie und Makromolekulare Chemie, Universität Jena, Humboldtstr. 10, 07743 Jena, Germany; b Institut für Anorganische und Analytische Chemie, Friedrich-Schiller-Universität, Jena, Humboldt-Str. 8, 07743 Jena, Germany

## Abstract

The title compound, C_12_H_9_BrN_2_OS_2_, was obtained by reacting 6-bromo­benzo[*d*]thia­zole-2-carbo­nitrile in *iso*-propanol with ethyl 2-mercapto-2-methyl­propano­ate at reflux temperature for several hours. The resulting di­methyl­oxyluciferin derivative shows partial double-bond character of the carbon–carbon bond between the two heterocyclic moieties [C—C = 1.461 (3) Å]. This double bond restricts rotation around this C—C axis, therefore leading to an almost planar mol­ecular structure [N—C—C—S torsion angle = 9.7 (3)°]. The five-membered thiazoline ring is not completely planar as a result of the bulky S atom [C—S—C—C torsion angle = 5.17 (12)°].

## Related literature

For the chemi- and bioluminescence of firefly luciferin and related compounds, see: Jung *et al.* (1975 ▶); White *et al.* (1961 ▶, 1979 ▶); Branchini *et al.* (2002 ▶). For structural modifications of firefly luciferin, see: Meroni *et al.* (2009 ▶); McCutcheon *et al.* (2012 ▶); Branchini *et al.* (2012 ▶); Würfel (2012 ▶). Luciferin and related structures are widely used in clinical and biochemical applications, see: Schäffer (1987*a* ▶,*b* ▶); Kricka (1988 ▶); Josel *et al.* (1994*a* ▶,*b* ▶); Shinde *et al.* (2006 ▶). For details of the synthetic procedure, see: Armarego & Chai (2009 ▶); Bardsley *et al.* (2009*a* ▶,*b* ▶); Würfel *et al.* (2012 ▶).
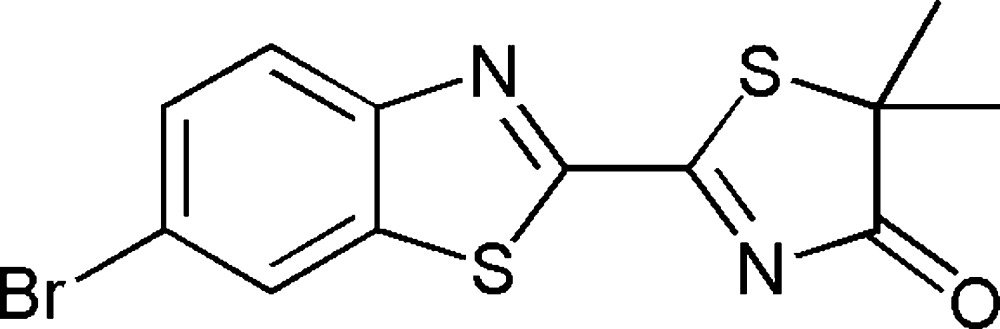


## Experimental

### 

#### Crystal data

C_12_H_9_BrN_2_OS_2_
*M* *_r_* = 341.24
Monoclinic, 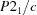

*a* = 12.8246 (3) Å
*b* = 11.9115 (3) Å
*c* = 8.5375 (2) Å
β = 99.735 (1)°
*V* = 1285.41 (5) Å^3^
*Z* = 4
Mo *K*α radiation
μ = 3.51 mm^−1^
*T* = 133 K
0.06 × 0.05 × 0.04 mm

#### Data collection

Nonius KappaCCD diffractometer
7856 measured reflections
2927 independent reflections
2676 reflections with *I* > 2σ(*I*)
*R*_int_ = 0.033

#### Refinement

*R*[*F* ^2^ > 2σ(*F* ^2^)] = 0.025
*wR*(*F* ^2^) = 0.065
*S* = 1.02
2927 reflections
165 parameters
H-atom parameters constrained
Δρ_max_ = 0.44 e Å^−3^
Δρ_min_ = −0.40 e Å^−3^

### 

Data collection: *COLLECT* (Nonius, 1998 ▶); cell refinement: *DENZO* (Otwinowski & Minor 1997 ▶); data reduction: *DENZO*; program(s) used to solve structure: *SHELXS97* (Sheldrick, 2008 ▶); program(s) used to refine structure: *SHELXL97* (Sheldrick, 2008 ▶); molecular graphics: *XP* in *SHELXTL/PC* (Sheldrick, 2008 ▶); software used to prepare material for publication: *SHELXL97*.

## Supplementary Material

Crystal structure: contains datablock(s) I, New_Global_Publ_Block. DOI: 10.1107/S1600536813031334/im2444sup1.cif

Structure factors: contains datablock(s) I. DOI: 10.1107/S1600536813031334/im2444Isup2.hkl

Supplementary material file. DOI: 10.1107/S1600536813031334/im2444Isup3.cml

Additional supplementary materials:  crystallographic information; 3D view; checkCIF report

## Figures and Tables

**Fig. 1.**
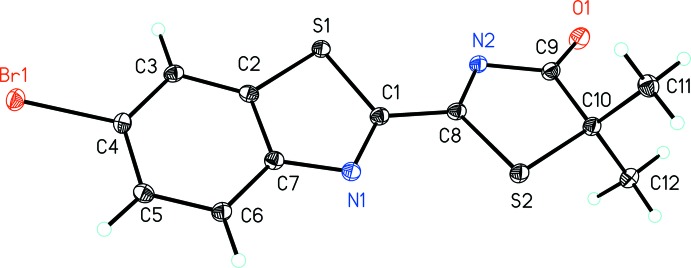
Molecular structure and numbering scheme of the title compound 1 showing displacement ellipsoids at the 40% probability level.

## supplementary crystallographic information

### 1. Comment

Heterocycles are widely used in nature for a great variety of purposes. One of the most interesting ones is bioluminescence which is defined as the chemically stimulated emission of light by living organisms. One prominent example of a bioluminescent organism is the firefly (*Photinus pyralis*), which employs the benzothiazole containing Luciferin for light emission (White *et al.*, 1961). One known inhibitor for this Luciferin-Luciferase reaction is the structurally close related dimethyloxyluciferin (Meroni *et al.*, 2009). This compound also shows a bright red fluorescence in the visible spectrum in the deprotonated state (Branchini *et al.*, 2002). Therefore investigations where conducted in our group focusing on modifications of the benzo[*d*]thiazol moiety of dimethyloxyluciferin (Würfel, 2012).

The title compound was synthesized by condensation of 6-bromobenzo[*d*]thiazole-2-carbonitrile with 2-mercapto-2-methylpropanoate. The Br—C bond length of 1.898 (2) Å (Br1—C4) is typical for a bromine atom bonded to an aromatic ring. The substituted benzo[*d*]-thiazol moiety builds up a planar structure, whereas the thiazoline ring is not exactly planar because of one *sp*^3^ carbon atom (C10). Since *sp*^3^ carbon atoms prefer smaller bond angles than *sp*^2^ carbons, C10 is pushed out of the thiazoline plane. The C—C bond, connecting the heterocyclic moieties shows a partial double bond character (C1—C8, 1.461 (3) Å). The sulfur atoms S1 and S2 are arranged *trans* in the crystal, thus providing the maximal distance from each other (4.3686 (7) Å). Despite of the double bond character of C1—C8 the heterocyclic moieties are not exactly coplanar with respect to each other. The torsion angles N1—C1—C8—S2 show a deviation of -9.7 (3)°.

### 2. Experimental

All chemicals were synthesized according to given literature or purchased from commercial sources. All solvents were purified and dried according to Armarego & Chai (2009). 3.46 g (14.5 mmol) 6-bromobenzo[*d*]thiazole-2-carbonitrile, 2.5 ml (approx. 17.4 mmol) ethyl 2-mercapto-2-methylpropanoate and 4.8 ml (34.8 mmol) triethylamine were refluxed in 20 ml of *iso*-propanol for 6 h. The product was recrystallized from ethanol yielding 70% (3.46 g, 10.1 mmol) pale yellow crystals. 6-Bromobenzo[*d*]thiazole-2-carbonitrile was prepared analog to Würfel *et al.* (2012). Ethyl 2-mercapto-2-methylpropanoate was prepared according to Bardsley *et al.* (2009*a*,*b*). Yellow single crystals of the title compound were obtained by dissolving the compound in ethanol at reflux temperature and after cooling to r. t. the closed vessel was left alone for several days. Elemental analysis calculated for C_12_H_9_BrN_2_OS_2_: C 42.24, H 2.66, Br 23.42, N 8.21, S 18.79; found: C 42.12, H 2.63, Br 23.51, N 8.11, S 19.00.

### 3. Refinement

All hydrogen atoms were set to idealized positions and were refined with *U*_iso_(H) = 1.2 *U*_eq_(C) (1.5 for methyl groups). Methyl groups were allowed to rotate but not to tip.

### Crystal data

**Table:**

C_12_H_9_BrN_2_OS_2_	*F*(000) = 680
*M**_r_* = 341.24	*D*_x_ = 1.763 Mg m^−^^3^
Monoclinic, *P*2_1_/*c*	Mo *K*α radiation, λ = 0.71073 Å
Hall symbol: -P 2ybc	Cell parameters from 7856 reflections
*a* = 12.8246 (3) Å	θ = 2.4–27.5°
*b* = 11.9115 (3) Å	µ = 3.51 mm^−^^1^
*c* = 8.5375 (2) Å	*T* = 133 K
β = 99.735 (1)°	Prism, colourless
*V* = 1285.41 (5) Å^3^	0.06 × 0.05 × 0.04 mm
*Z* = 4	

### Data collection

**Table:**

Nonius KappaCCD diffractometer	2676 reflections with *I* > 2σ(*I*)
Radiation source: fine-focus sealed tube	*R*_int_ = 0.033
Graphite monochromator	θ_max_ = 27.5°, θ_min_ = 2.4°
phi– + ω–scan	*h* = −16→16
7856 measured reflections	*k* = −15→15
2927 independent reflections	*l* = −11→11

### Refinement

**Table:**

Refinement on *F*^2^	Primary atom site location: structure-invariant direct methods
Least-squares matrix: full	Secondary atom site location: difference Fourier map
*R*[*F*^2^ > 2σ(*F*^2^)] = 0.025	Hydrogen site location: inferred from neighbouring sites
*wR*(*F*^2^) = 0.065	H-atom parameters constrained
*S* = 1.02	*w* = 1/[σ^2^(*F*_o_^2^) + (0.0282*P*)^2^ + 1.1743*P*] where *P* = (*F*_o_^2^ + 2*F*_c_^2^)/3
2927 reflections	(Δ/σ)_max_ = 0.001
165 parameters	Δρ_max_ = 0.44 e Å^−^^3^
0 restraints	Δρ_min_ = −0.40 e Å^−^^3^

### Special details

**Table:**

Geometry. All e.s.d.'s (except the e.s.d. in the dihedral angle between two l.s. planes) are estimated using the full covariance matrix. The cell e.s.d.'s are taken into account individually in the estimation of e.s.d.'s in distances, angles and torsion angles; correlations between e.s.d.'s in cell parameters are only used when they are defined by crystal symmetry. An approximate (isotropic) treatment of cell e.s.d.'s is used for estimating e.s.d.'s involving l.s. planes.
Refinement. Refinement of *F*^2^ against ALL reflections. The weighted *R*-factor *wR* and goodness of fit *S* are based on *F*^2^, conventional *R*-factors *R* are based on *F*, with *F* set to zero for negative *F*^2^. The threshold expression of *F*^2^ > σ(*F*^2^) is used only for calculating *R*-factors(gt) *etc*. and is not relevant to the choice of reflections for refinement. *R*-factors based on *F*^2^ are statistically about twice as large as those based on *F*, and *R*- factors based on ALL data will be even larger.

### Fractional atomic coordinates and isotropic or equivalent isotropic displacement parameters (Å^2^)

**Table:**

	*x*	*y*	*z*	*U*_iso_*/*U*_eq_	
Br1	0.858415 (15)	0.276647 (17)	−0.06700 (2)	0.02385 (8)	
S1	0.46626 (4)	0.21606 (4)	0.14052 (6)	0.01968 (11)	
S2	0.26944 (4)	0.47143 (4)	0.31410 (6)	0.02169 (12)	
O1	0.11449 (13)	0.20363 (13)	0.3753 (2)	0.0295 (4)	
N1	0.48242 (12)	0.42814 (14)	0.21789 (19)	0.0197 (3)	
N2	0.26810 (13)	0.25109 (15)	0.2854 (2)	0.0206 (3)	
C1	0.42214 (15)	0.34012 (16)	0.2154 (2)	0.0190 (4)	
C2	0.57634 (15)	0.28879 (16)	0.1035 (2)	0.0180 (4)	
C3	0.66043 (15)	0.24949 (16)	0.0334 (2)	0.0188 (4)	
H3A	0.6623	0.1748	−0.0049	0.023*	
C4	0.74046 (15)	0.32508 (17)	0.0230 (2)	0.0188 (4)	
C5	0.73963 (15)	0.43642 (17)	0.0770 (2)	0.0202 (4)	
H5A	0.7973	0.4850	0.0691	0.024*	
C6	0.65474 (15)	0.47491 (17)	0.1416 (2)	0.0208 (4)	
H6A	0.6525	0.5505	0.1765	0.025*	
C7	0.57210 (15)	0.40109 (16)	0.1549 (2)	0.0188 (4)	
C8	0.32045 (15)	0.34245 (16)	0.2709 (2)	0.0189 (4)	
C9	0.17367 (16)	0.27547 (16)	0.3418 (2)	0.0211 (4)	
C10	0.14997 (15)	0.40174 (16)	0.3551 (2)	0.0195 (4)	
C11	0.05518 (16)	0.43242 (19)	0.2281 (2)	0.0250 (4)	
H11B	−0.0072	0.3904	0.2471	0.037*	
H11C	0.0708	0.4134	0.1228	0.037*	
H11D	0.0412	0.5131	0.2330	0.037*	
C12	0.13018 (16)	0.42953 (18)	0.5223 (2)	0.0231 (4)	
H12B	0.0677	0.3886	0.5433	0.035*	
H12C	0.1183	0.5104	0.5307	0.035*	
H12D	0.1919	0.4073	0.6002	0.035*	

### Atomic displacement parameters (Å^2^)

**Table:**

	*U*^11^	*U*^22^	*U*^33^	*U*^12^	*U*^13^	*U*^23^
Br1	0.02181 (11)	0.02233 (12)	0.02884 (12)	0.00282 (7)	0.00841 (8)	−0.00194 (8)
S1	0.0206 (2)	0.0135 (2)	0.0256 (2)	−0.00276 (17)	0.00599 (19)	−0.00213 (18)
S2	0.0223 (2)	0.0131 (2)	0.0311 (3)	−0.00125 (17)	0.00843 (19)	−0.00089 (19)
O1	0.0320 (8)	0.0187 (7)	0.0420 (9)	−0.0057 (6)	0.0182 (7)	−0.0017 (7)
N1	0.0204 (8)	0.0157 (8)	0.0237 (8)	−0.0015 (6)	0.0059 (6)	−0.0001 (6)
N2	0.0204 (8)	0.0160 (8)	0.0263 (8)	−0.0018 (6)	0.0062 (7)	−0.0031 (7)
C1	0.0208 (9)	0.0163 (9)	0.0196 (9)	0.0010 (7)	0.0024 (7)	−0.0003 (7)
C2	0.0200 (9)	0.0158 (9)	0.0177 (9)	−0.0029 (7)	0.0019 (7)	0.0015 (7)
C3	0.0218 (9)	0.0138 (9)	0.0204 (9)	0.0018 (7)	0.0023 (7)	−0.0015 (8)
C4	0.0183 (8)	0.0198 (9)	0.0187 (9)	0.0023 (7)	0.0043 (7)	0.0016 (8)
C5	0.0212 (9)	0.0175 (9)	0.0220 (9)	−0.0030 (7)	0.0035 (7)	0.0009 (7)
C6	0.0224 (9)	0.0152 (9)	0.0258 (10)	−0.0004 (7)	0.0064 (8)	−0.0014 (8)
C7	0.0203 (9)	0.0157 (9)	0.0202 (9)	0.0006 (7)	0.0026 (7)	0.0007 (7)
C8	0.0216 (9)	0.0139 (9)	0.0209 (9)	0.0001 (7)	0.0024 (7)	−0.0003 (7)
C9	0.0231 (9)	0.0180 (10)	0.0229 (10)	−0.0001 (7)	0.0058 (8)	−0.0019 (8)
C10	0.0204 (9)	0.0158 (9)	0.0235 (9)	−0.0007 (7)	0.0067 (7)	−0.0001 (7)
C11	0.0232 (9)	0.0288 (11)	0.0226 (10)	0.0006 (8)	0.0031 (8)	0.0017 (8)
C12	0.0255 (10)	0.0225 (10)	0.0221 (10)	0.0031 (8)	0.0064 (8)	−0.0012 (8)

### Geometric parameters (Å, º)

**Table:**

Br1—C4	1.8985 (19)	C3—H3A	0.9500
S1—C2	1.730 (2)	C4—C5	1.405 (3)
S1—C1	1.742 (2)	C5—C6	1.379 (3)
S1—S2	4.3686 (7)	C5—H5A	0.9500
S2—C8	1.734 (2)	C6—C7	1.396 (3)
S2—C10	1.8276 (19)	C6—H6A	0.9500
O1—C9	1.210 (3)	C9—C10	1.542 (3)
N1—C1	1.301 (3)	C10—C12	1.528 (3)
N1—C7	1.387 (2)	C10—C11	1.530 (3)
N2—C8	1.296 (3)	C11—H11B	0.9800
N2—C9	1.407 (3)	C11—H11C	0.9800
C1—C8	1.461 (3)	C11—H11D	0.9800
C2—C3	1.399 (3)	C12—H12B	0.9800
C2—C7	1.412 (3)	C12—H12C	0.9800
C3—C4	1.379 (3)	C12—H12D	0.9800
			
C2—S1—C1	88.17 (9)	N1—C7—C2	114.75 (17)
C2—S1—S2	104.45 (7)	C6—C7—C2	120.10 (18)
C1—S1—S2	16.82 (6)	N2—C8—C1	121.36 (18)
C8—S2—C10	89.82 (9)	N2—C8—S2	120.25 (15)
C8—S2—S1	18.27 (7)	C1—C8—S2	118.38 (15)
C10—S2—S1	107.45 (6)	O1—C9—N2	123.07 (18)
C1—N1—C7	109.63 (17)	O1—C9—C10	122.21 (19)
C8—N2—C9	110.44 (17)	N2—C9—C10	114.71 (17)
N1—C1—C8	122.68 (18)	C12—C10—C11	111.89 (16)
N1—C1—S1	117.44 (15)	C12—C10—C9	110.22 (17)
C8—C1—S1	119.88 (15)	C11—C10—C9	108.90 (17)
C3—C2—C7	121.53 (17)	C12—C10—S2	110.94 (14)
C3—C2—S1	128.47 (15)	C11—C10—S2	110.32 (14)
C7—C2—S1	109.99 (15)	C9—C10—S2	104.29 (13)
C4—C3—C2	116.41 (18)	C10—C11—H11B	109.5
C4—C3—H3A	121.8	C10—C11—H11C	109.5
C2—C3—H3A	121.8	H11B—C11—H11C	109.5
C3—C4—C5	123.22 (18)	C10—C11—H11D	109.5
C3—C4—Br1	118.70 (15)	H11B—C11—H11D	109.5
C5—C4—Br1	118.07 (15)	H11C—C11—H11D	109.5
C6—C5—C4	119.70 (18)	C10—C12—H12B	109.5
C6—C5—H5A	120.2	C10—C12—H12C	109.5
C4—C5—H5A	120.2	H12B—C12—H12C	109.5
C5—C6—C7	118.98 (18)	C10—C12—H12D	109.5
C5—C6—H6A	120.5	H12B—C12—H12D	109.5
C7—C6—H6A	120.5	H12C—C12—H12D	109.5
N1—C7—C6	125.15 (18)		
			
C2—S1—S2—C8	−164.7 (2)	C3—C2—C7—C6	−2.3 (3)
C1—S1—S2—C8	−149.8 (3)	S1—C2—C7—C6	177.93 (15)
C2—S1—S2—C10	179.56 (9)	C9—N2—C8—C1	179.51 (17)
C1—S1—S2—C10	−165.6 (2)	C9—N2—C8—S2	−1.7 (2)
C7—N1—C1—C8	−178.67 (17)	N1—C1—C8—N2	−171.43 (19)
C7—N1—C1—S1	0.8 (2)	S1—C1—C8—N2	9.2 (3)
C2—S1—C1—N1	−1.39 (16)	N1—C1—C8—S2	9.7 (3)
S2—S1—C1—N1	−167.0 (3)	S1—C1—C8—S2	−169.70 (11)
C2—S1—C1—C8	178.06 (16)	C10—S2—C8—N2	−2.53 (17)
S2—S1—C1—C8	12.46 (13)	S1—S2—C8—N2	−167.5 (3)
C1—S1—C2—C3	−178.28 (19)	C10—S2—C8—C1	176.35 (16)
S2—S1—C2—C3	−174.01 (16)	S1—S2—C8—C1	11.38 (12)
C1—S1—C2—C7	1.52 (15)	C8—N2—C9—O1	−174.9 (2)
S2—S1—C2—C7	5.78 (14)	C8—N2—C9—C10	6.1 (2)
C7—C2—C3—C4	2.4 (3)	O1—C9—C10—C12	54.4 (3)
S1—C2—C3—C4	−177.87 (15)	N2—C9—C10—C12	−126.62 (18)
C2—C3—C4—C5	−0.7 (3)	O1—C9—C10—C11	−68.7 (3)
C2—C3—C4—Br1	178.80 (14)	N2—C9—C10—C11	110.29 (19)
C3—C4—C5—C6	−1.2 (3)	O1—C9—C10—S2	173.56 (18)
Br1—C4—C5—C6	179.35 (15)	N2—C9—C10—S2	−7.5 (2)
C4—C5—C6—C7	1.3 (3)	C8—S2—C10—C12	123.84 (15)
C1—N1—C7—C6	−178.89 (19)	S1—S2—C10—C12	128.72 (12)
C1—N1—C7—C2	0.5 (2)	C8—S2—C10—C11	−111.60 (15)
C5—C6—C7—N1	179.72 (18)	S1—S2—C10—C11	−106.71 (13)
C5—C6—C7—C2	0.3 (3)	C8—S2—C10—C9	5.19 (14)
C3—C2—C7—N1	178.30 (17)	S1—S2—C10—C9	10.08 (14)
S1—C2—C7—N1	−1.5 (2)		
